# Massachusetts Veterinary Association

**Published:** 1892-12

**Authors:** Austin Peters

**Affiliations:** Secretary


*Massachusetts Veterinary Association.* Special meeting of the Massachusetts Veterinary Association held at 19 Boylston Place, Boston, Wednesday evening, Sept. 14, 1892, at 7:30 o'clock.

President L. H. Howard in the chair. Members present: Drs. Bryden,

Blackwood, Becket, Burr, Hadcock, Howard, Winchester, Hitchcock, Parker, Stinson, and the Secretary. Honorary member, Dr. Stickney.

The meeting was held to make final arrangements for the meeting of the United States Veterinary Medical Association in Boston, the following week.

The reports of the various committees having charge of the work were received, and it was shown that every detail had been carefully attended to.

The meeting then adjourned.

AUSTIN PETERS, *Secretary*.

*Massachusetts Veterinary Association.* Regular meeting of the Massachusetts Veterinary Association held at 19 Boylston Place, Boston, Wednesday evening, Oct. 26, 1892, at 7.30 o'clock.

President L. H. Howard in the chair. Members present: Drs. Bryden, Blackwood, Burr, Hadcock, Howard, Marshall, Osgood, Winchester. Winslow, Parker, La Baw, Stinson, and the Secretary. Honorary member, Dr. Stickney, Visitors: Messrs. Cronin and Dowd, students at the Harvard Veterinary School.

Records of a regular June meeting, and a special meeting held in September, read and accepted. Unfinished business, none

Reports of committees: Dr. Winchester reported for the committee appointed to arrange for entertaining the United States Veterinary Medical Association during its meeting in Boston, last September. Moved by Dr. Osgood, and seconded by Dr. Blackwood, that the report be accepted. Carried. Moved by Dr. Osgood, seconded by Dr. Blackwood, that the surplus from the entertainment fund be paid into the Association's treasury. Carried.

Admission of members: Names of Drs. E. P. McKenna and Wilbert Soule balloted upon. Twelve votes cast for each, all in the affirmative. They were therefore elected.

Application for membership: The application and credentials of W. M. Balmer, M.D.V (Harv.), were reported favorably upon by the Executive Committee. Dr. Balmer's name is laid on the table for a month, under the rules.

New business: Dr. Winchester called the attention of members of the Association to the first annual announcement of the "National Veterinary School" in Washington, many members of the faculty being veterinarians, who are either members of the U.S.V.M.A., or connected with the United States Bureau of Animal Industry, or both.

He also called the attention of the members of the Association to the new requirements adopted by the U.S.V.M.A., at its last meeting, in Boston, that hereafter that Association admits to membership only graduates of schools giving a three years' graded course of instruction.

The amendment to the U.S.V.M.A. Constitution was adopted unanimously, that is, there was not a dissenting vote, and silence gives assent, and yet there were men sitting in the hall who had the announcement of the new school in their pockets and yet did not say a word. Dr. Winchester thinks it one of the most slanderous affairs ever thrown in the face of our profession, and although it may do no good to say anything, yet silence is acknowledgment, and it is our duty to decry this institution. We ought to act to-night; we ought to show our disapprobation to the proper United States authorities, and we ought also to publicly censure the officials of the Bureau of Animal Industry for their participation in the matter. Dr. Winchester then read the resolutions of the Philadelphia Veterinary Society at its meeting September 27th. These resolutions cover the ground pretty well, but they might have been even more emphatic. He also read Dr. Cooper Curtice's open letter to Hon. Jerry Rusk. Dr. Winchester thinks that we as an Association ought to make our protest on the same basis as the Philadelphia resolutions and Dr. Curtice's letter. Furthermore, as citizens of the United States, voters, and tax payers. as well as members of this Association, we have a right to object to public officials using their positions in such a way.

Moved by Dr. Parker that the chair appoint a committee of three to draw resolutions protesting against the formation of a "National" school with a two years' course, particularly after its founders had placed themselves on record as favoring a three years' graded course of study. Dr Peters amends the motion, that the committee memorialize Hon. Jerry Rusk, and also draw resolutions censuring the action of the Bureau of Animal Industry, wherein their course is reprehensible as public officials. Seconded by Dr. Winchester. Accepted by Dr. Parker.

Dr. Bryden thinks we ought to memorialize the faculty of this school, and not attack them in an arbitrary way We ought to do it kindly and harmoniously.

Dr. Howard thinks that other schools have been started with a two years' course, and have had officials of the Bureau of Animal Industry as teachers, and our course must be consistent. Motion then put and carried without a dissenting voice.

Dr Howard then said that our course must be consistent and what we send must be something nobody can go behind. He then appointed Drs. Parker, Bryden and Peters on the committee. All refuse to accept. President Howard then appointed Drs. Parker, Bryden and Winchester as a committee. Motion then made by Dr. Peters that the committee report at the next meeting. Seconded by Dr. Winslow. Carried.

Reports of cases: Dr. Winslow, of Rockland, has procured a case of springhalt which is now at the Harvard Veterinary Hospital, where members can see him. He was to have been brought down town for the members of the Association to see to-night, but he has become incapacitated in some way and could not come.

Dr. Bryden reported having examined him to see if it was worth while to attempt treating him. He says the horse's hocks are deformed; he is old, and his reparative powers are well gone, and he doubts if treatment will do any good, but is willing to try.

Dr. Winslow first saw the animal two weeks ago. He does not show well marked signs of springhalt when quiet, but if started up suddenly for a little way, then stopped for a moment, and then started again, he shows it in one leg almost as much as the old black horse that the Association killed a year ago last July.

Dr. Howard then read a letter from Mr. T. L. Bolton, of Clark University, in which, among other things, said he was willing to investigate this case as he did the last one.

Dr. Bryden reports a conversation he had with a professor* at the Harvard Medical School. He told the professor that he thought that springhalt was due to a pheripheral disturbance, and that they did not agree with him at Clark University. The professor told Dr. Bryden that changes in the spinal cord, such as he described, were not infrequently met with in various affections in the human, such as elephantiasis, schleroderma, lupus, etc.

*The professor's name withheld at Dr. Bryden's request.

Dr. Howard says Dr. Winslow gives the Association this horse ; he is our property, and we shall insist that a full report of the investigation of the case first appears in the veterinary journals.

Dr. Bryden says it is not a good case to treat, and Dr. Osgood agrees that it may not be.

Dr Howard thinks that the foot is not as much contracted as the little black's was. Motion made by Dr. Winchester that we accept the horse, and are glad to keep him at the Harvard Veterinary School for awhile, and that Mr. Bolton be invited to come and experiment with him. Seconded by Dr. Blackwood. Carried. Motion made by Dr. Osgood that we give Dr. Winslow a vote of thanks for the horse, and credit him on the Association's books with a sum equal to the expense he has gone to in the matter. Seconded by Dr. Winchester. Carried.

Dr. Bryden showed two very large ergots from back of the fetlocks of a grey horse. In his opinion the horn here developed in excess on account of the feet being imperfectly nourished.

Dr. Winchester showed a specimen of aneurism in a branch of the anterior mesenteric artery, due to the strongylus armatus. This artery contained a chain of hrombi due to this cause, and free strongyles were formed in the posterior aorta. The animal from which the specimens were obtained was a mare about twenty years old from which several fast colts have been raised. Dr Winchester had known her for nine or ten years. Several years ago she was in poor flesh while breeding, and had to be given twelve or sixteen quarts of oats daily to keep her in condition. Her powers of assimilation did not seem to be what they should. Last summer she was at Lowell with a sucking foal. The owner started to take her to Haverhill, but she only went as far as Lawrence, when she was taken sick, and was finally destroyed. Principal sympton loss of power in hind legs, also had hæmaturea at first, but recovered from this condition before being killed. The only other abnormality than those already described, upon post-mortem, was a serous cyst on the outside of one kidney. Dr. Winchester never knew of this mare having colic, but she evidently did have loss of nutrition.

Dr. Peters showed a small tumor removed from a horse's breast, which proved to be a dermoid cyst containing hairs and what appeared to be broken down epithelial cells, when it was cut open.

Dr. Howard reported a case of scrotal hernia in a gelding. The animal was a bay gelding, six years old, driven every day since his arrival in the city twelve days previously. No history of previous trouble of any kind. On the seventh day the driver thought he was driving a stallion, because of an enlargement noticed by him in the inguinal region. After noticing the swelling, he was driven five miles farther, and upon returning home, the animal laid down and was quiet for three hours. At the end of that time slight pain was noticed, and a veterinarian called. He made a diagnosis of hernia, and applied a truss made of a piece of blanket, and a favorable prognosis was given. Dr. Howard was called fifteen hours later, found a swelling the size of a peck basket, constant pain, animal pulseless, vacant stare, temperature 102½. Advised his destruction. Post-mortem showed seven feet of intestine passed through the inguinal ring, strangulated.

Dr. La Baw remembered seeing a case of scrotal hernia in a gelding in New York. Dr. Osgood once saw a three-year-old colt with a scrotal hernia, four months after castration. He threw the colt, reduced the hernia, applied clamps to the scrotum and the animal made a complete recovery.

Dr. Parker reported a case of pneumo-entertis in a horse, the urine being so thick and sticky that it could hardly be voided. It shows no albumen, but contains an excess of salts.

Dr. Bryden said that after the outbreak of so-called spinal meningitis in 1873, the urine was in this condition.

Dr. Parker says his case is improving on dilute hydrochloric acid.

Meeting then adjourned.

AUSTIN PETERS, *Secretary.*

